# Using health markets to improve access to medicines: three case studies

**DOI:** 10.1186/s40545-016-0067-5

**Published:** 2016-05-06

**Authors:** Zubin Shroff, Maryam Bigdeli, Zaheer-Ud-Din Babar, Anita Wagner, Abdul Ghaffar, David H Peters

**Affiliations:** Alliance for Health Policy and Systems Research, World Health Organization, Geneva, Switzerland; Health Systems Governance, Policy and Aid Effectiveness, World Health Organization, Geneva, Switzerland; University of Auckland, Auckland, New Zealand; Harvard Medical School, Boston, USA; Johns Hopkins Bloomberg School of Public Health, Baltimore, USA

## Abstract

This editorial introduces a series of case studies that together highlight the use of health market interventions to improve access to medicines in low-and-middle income countries (LMICs). It underscores the added value of using a systems approach for a holistic understanding of how these interventions interact with the rest of the health system and the intended and unintended consequences that result. It goes on to summarize key findings from each of the studies and concludes with lessons for decision-makers on the design and implementation of market based interventions in LMIC health systems

## Editorial

Despite decades of implementation of national medicines policies, medicines access, affordability, and use are still problematic in many low and middle-income countries (LMICs). Medicines account for a high proportion of out-of-pocket (OOP) expenses in many LMICs and challenges exist to ensure equitable access in the face of geographic, economic and cultural barriers [2, 3]. Ineffective regulatory systems, inadequate knowledge of disease and medicines both on the part of providers (many of whom are unlicensed) and patients, and wrong incentives are some of the reasons for widespread inappropriate use of medicines [6].

Markets have played an increasingly important role in the health systems of LMICs over recent years, particularly in drug development and in the delivery of related products and services. A thorough understanding of health market systems is needed to improve access to and use of medicines [6]. New laws and regulations, training of providers, and dissemination of information to relevant stakeholders are some of the interventions that have been implemented. These interventions have been effective in engaging with health market actors, including pharmaceutical companies, drug distributers, pharmacies, providers and consumers and counteracting well known market failures [6]. Yet strategies that depend on single interventions often fail. This is because they do not adequately anticipate and account for complex interactions among the range of stakeholders mentioned above.

A systems approach to medicines is thus needed. A systems approach examines medicines within the existent complexity of a health system, and sheds light on how ‘interventions in the pharmaceutical sector influence the rest of the health system and vice versa’. It explicitly recognizes that the development, production, procurement, distribution, prescription, dispensation and use of medicines depend on the dynamic interaction of various health system components [6]. It can provide new insights on why interventions succeed or fail, how they need to change over time, and helps identify inputs, processes, outputs and outcomes in need of impactful monitoring and evaluation. Using a systems perspective can thus overcome limitations of designing and evaluating narrowly-targeted interventions and can provide vital information to design and implement system-focused strategies [6].

Each of the three case studies that comprise this series uses a systems approach to examine interventions that have sought to improve access to medicines in LMICs through the innovative use of health markets.

The first paper by Rutta et al. [4] reflects on the experience of Tanzania’s Accredited Drug Dispensing Outlet (ADDO) program, a public-private partnership that seeks to increase access to high-quality medicines at affordable prices in areas lacking registered pharmacies through engaging private retail drug shops. The authors examine the program’s continued evolution in bringing together training, business incentives, regulation, institution building and information sharing. It also describes how the multi-pronged intervention strategy moved health system building blocks towards better access to medicines. The paper identifies early and continuous engagement of stakeholders at all levels as a key element of the program’s success.

Van Olmen et al. [5] examine how the innovative MoPoTsyo peer educator program for diabetes patients has enabled access to diabetes care in Cambodia, and the stakeholder perceptions surrounding this approach. They describe the evolution of the program from the self-management of diabetes to a multi-component intervention that simultaneously enabled access to affordable medicines, provided services, and fostered integration of peer educators with the country’s public health system. The authors conclude that a combination of empowering patients, strong organizational ability, engaging health system actors at all levels, and simultaneous action on multiple barriers to care were central to achieving positive program outcomes.

The final paper, by Ashigbie et al. [1], discusses the challenges posed by medicines reimbursement policies and management implemented under Ghana’s National Health Insurance Scheme (NHIS) and the consequences these have on public and private providers and patients. Using key informant interviews to understand accreditation, drug reimbursement, medicines selection and purchasing policies, and utilization, they found that while utilization had increased under the NHIS, several factors undermined the NHIS’ effectiveness in improving affordable access to needed medicines. These included delayed and low reimbursement for medicines and inadequate regulatory arrangements, which contributed to private providers dropping out of the scheme.

There are several lessons that emerge from the case studies. First, they highlight the importance of identifying neglected stakeholders (e.g. drug shops in Tanzania, peer educators in Cambodia) in addressing market failures in health markets. All three cases demonstrate the need for early and continuous engagement of a wide range of stakeholders. The Ghana study is an excellent example of the consequences of program implementation that does not adequately address key stakeholder concerns [6]. The case studies also bring to the fore the importance of putting in place dynamic, multi-component interventions. Interventions were dynamic because they evolved over time in response to changed incentive structures, new technology and new information; and they comprised multiple components or addressed different elements of the health system. Evaluations assessed potential unintended consequences, which were successfully managed in Tanzania and Cambodia, and are now of particular importance in Ghana as the country continues to move forward on the path to universal health coverage [2, 6]. Finally, the case studies highlight the importance of strong institutional mechanisms to underpin implementation of change, whether in the form of training providers, creating effective regulatory schemes, developing information systems, or strengthening supply chains. Health system strengthening efforts towards universal health coverage seek to reach all populations with an expanding range of needed, high-quality services without causing financial hardship. This also represents a major opportunity to design and put in place reforms that are built on the engagement of multiple stakeholders, supported by appropriate institutional arrangements, and informed by evidence. Such evidence is generated through monitoring intended and unintended consequences of changes in health systems.
